# Biotechnological Advances in Pharmacognosy and In Vitro Manipulation of *Pterocarpus marsupium* Roxb.

**DOI:** 10.3390/plants11030247

**Published:** 2022-01-18

**Authors:** Anees Ahmad, Naseem Ahmad, Mohammad Anis, Mohammad Faisal, Abdulrahman A. Alatar, Eslam M. Abdel-Salam, Ram Pratap Meena, Iyyakkannu Sivanesan

**Affiliations:** 1 Plant Biotechnology Laboratory, Department of Botany, Aligarh Muslim University, Aligarh 202002, India; aneesqadriamu@gmail.com (A.A.); naseembot@gmail.com (N.A.); anism1@rediffmail.com (M.A.); 2 Pharmacognosy Laboratory, Drug Standardization Research Institute, Central Council for Research in Unani Medicine, Ministry of AYUSH, Government of India, New Delhi 110058, India; 3 Department of Botany & Microbiology, College of Science, King Saud University, P.O. Box 2455, Riyadh 11451, Saudi Arabia; faisalm15@yahoo.com (M.F.); aalatar@ksu.edu.sa (A.A.A.); eabdelsalam@ksu.edu.sa (E.M.A.-S.); 4 Chemistry Laboratory, Central Council for Research in Unani Medicine (CCRUM), Ministry of AYUSH, New Delhi 110058, India; ccrumdsr2017@gmail.com; 5 Department of Bioresources and Food Science, Institute of Natural Science and Agriculture, Konkuk University, 1 Hwayang-dong, Gwangjin-gu, Seoul 05029, Korea

**Keywords:** biotechnological tools, DNA barcoding, ethnomedicine, in vitro culture, genetic improvement

## Abstract

Trees are vital resources for economic, environmental, and industrial growth, supporting human life directly or indirectly through a wide variety of therapeutic compounds, commodities, and ecological services. *Pterocarpus marsupium* Roxb. (Fabaceae) is one of the most valuable multipurpose forest trees in India and Sri Lanka, as it is cultivated for quality wood as well as pharmaceutically bioactive compounds, especially from the stem bark and heartwood. However, propagation of the tree in natural conditions is difficult due to the low percentage of seed germination coupled with overexploitation of this species for its excellent multipurpose properties. This overexploitation has ultimately led to the inclusion of *P. marsupium* on the list of endangered plant species. However, recent developments in plant biotechnology may offer a solution to the overuse of such valuable species if such advances are accompanied by technology transfer in the developing world. Specifically, techniques in micropropagation, genetic manipulation, DNA barcoding, drug extraction, delivery, and targeting as well as standardization, are of substantial concern. To date, there are no comprehensive and detailed reviews of *P. marsupium* in terms of biotechnological research developments, specifically pharmacognosy, pharmacology, tissue culture, authentication of genuine species, and basic gene transfer studies. Thus, the present review attempts to present a comprehensive overview of the biotechnological studies centered on this species and some of the recent novel approaches for its genetic improvement.

## 1. Introduction

Forest trees provide valuable resources for economic, environmental, and industrial development. Indeed, these plants sustain human life directly or indirectly, which supply a wide range of goods and ecological services essential for survival and prosperity. The medicinal plants used in traditional medicine all over the world are a potentially rich source of therapeutic compounds. Population increase along with rapid technological advances are putting tremendous pressure on natural genetic resources, especially in developing countries, where such resources are rapidly declining, and more species face extinction [1]. The more than 70 species in the pantropical genus *Pterocarpus* (Fabaceae) are also faced with development pressure [2]. The use of different plant parts in *Pterocarpus* spp. to treat illnesses since ancient times has been well documented [3,4,5,6,7,8]. The species has received much attention in experimental studies because of the growing evidence of potential bioactivities. The increasing demand for wood has led to unsustainable harvesting from wild sources of three species of *Pterocarpus*, namely *Pterocarpus marsupium*, *P. santalinus*, and *P. indicus*, which are now recognized as threatened species [9]. Besides the pharmaceutical value of *Pterocarpus* species, the wood is also valuable for bridge and boat building, as well as small-scale construction materials, plywood, veneer, and specialty wood for musical instruments [10].

The increase in public awareness of phytochemical-based drugs and the rapid growth of plant-based pharmacological industries have led to a greater demand for, and overexploitation of, natural flora. An increase in subsistence or non-commercial harvesting, as well as recent climatic changes have harmed many plant species, including *Pterocarpus* spp. One possible biotechnical response to the decline of some species is micropropagation, which capitalizes on the totipotent nature of plant cells [11]. In vitro propagation through tissue culture plays an important role in mass multiplication, plant improvement, plant breeding, regeneration of elite or superior clones, exchange of planting materials, secondary metabolites production, and germplasm conservation [12,13]. However, conventional propagation practice is time-consuming and labor-intensive and requires the availability of many plants for plantation or afforestation. Notwithstanding these challenges, plant cell, tissue, and organ culture techniques will become increasingly important in the cultivation of medicinal, as well as aromatic, plants by providing healthy and disease-free planting stock that can be widely utilized in commercial propagation and reforestation programs [14,15]. The information on actual genetic structure and the cryptic number of the differentiated genetic resources are valuable aids not only for developing in vitro regeneration protocols but also for the conservation of these medicinal plants. Many adulterants of *Pterocarpus* plant materials are available in the market, and they affect the efficacy of the drug; in some cases, these adulterants might be toxic and can prove to be lethal. A DNA barcoding technique can be effectively utilized to characterize and authenticate *Pterocarpus* and the detection of adulterants [16]. This technique is an alternative for rapid and robust species identification of genuine plant materials for the herbal drug industry [17].

*P. marsupium* propagates only by seed; the germination rate has been reported to be less than 30%, apparently because of the hard fruit coat coupled with poor viability and pod setting [18]. The mature fruits are harvested from the trees in April and May or before they drop to the ground. Pathogenic infections of fallen fruit also affect the germination rate under natural conditions [19]. Ahmad [20] recommended freshly collected seeds as a good planting source for obtaining healthy plantlets. The oleo-resin exudates of this species contain several unique active constituents, including vijyayosin, pterosupin, marsupsin, and pterostilbene, all of which show a wide range of pharmacological activity [21]. In addition, the National Medicinal Plant Board (NMPB) of India has estimated that the annual trade value of *P. marsupium* is approximately 300–500 metric tons per year and that each mature tree (10–15 years) produces approximately 0.5–0.6 tons of dry heartwood, valued at US $ 1200–1500 for each mature tree in the international market. Due to the aforementioned increased interest in recent years in the pharmaceutical, as well as the economic value of *P. marsupium*, the government of India has begun to encourage programs for large-scale cultivation and conservation of this species. The high demand for oleo-resin and wood, unsustainable harvesting practices, anthropogenic threats, and lack of regeneration, have together resulted in the rapid decline of natural populations. Furthermore, illegal harvesting of oleo-resin by damaging or wounding the wood can cause the trees to be susceptible to pests or diseases. Thus, uncontrolled extraction of oleo-resin could lead to adult mortality in combination with fragmentation and a low regeneration rate, threatening the persistence of the species.

Despite the enormous ethnobotanical value of *P. marsupium*, there is limited information on its genetic structure. Data on intraspecific relatedness is vital for selecting the best genotypes for plant breeding, effective population management, and conservation of germplasm. Given that genetic diversity allows populations to adapt to changing environments, the investigation of the genetic diversity of *P. marsupium* is not only important for species conservation, but also for the development and utilization of germplasm for improvement of this valuable but threatened medicinal plant. *P. marsupium* is a highly valuable medicinal plant but is a threatened species in India. Efforts for the conservation and propagation of this tree species will ultimately lead to the development of policies to make the species available for general use at a low cost. In view of these facts, it is of paramount importance to develop biotechnological techniques that ensure rapid propagation, multiplication, and authentication of the species for optimal germplasm conservation. With this goal in mind, we review the biotechnological advances made to date in *P. marsupium* concerning pharmacognosy, in vitro culture, multiplication, and genetic improvement.

## 2. Botanical Description of *P. marsupium*

**Figure 1:**
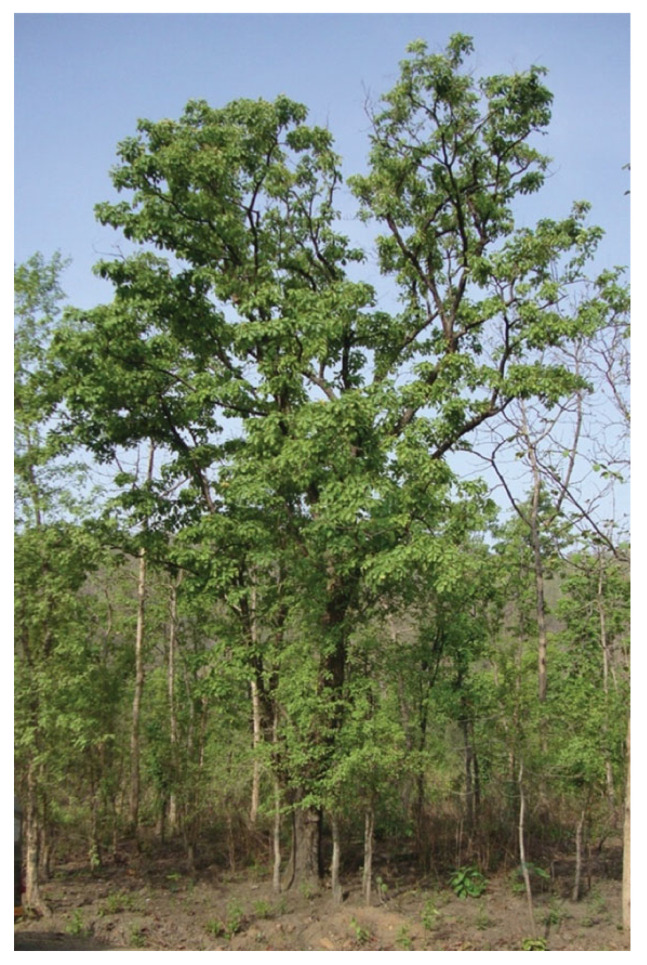
*Pterocarpus marsupium* Roxb. (22°23′25″ N and 84°21′44″ E, Chhattisgarh state of India).

*Pterocarpus marsupium* Roxb., a legume, commonly known as “Bijasal” or “Indian Kino Tree,” (Figure 1) is a valuable multipurpose, as well as industrially important, forest tree. Ahmad and Anis [22] suggested that it is a potential herbal drug yielding tree in India. Generally, the tree is found in dry mixed deciduous tropical forests and flourishes in the open sun under “80” to “200” cm moderate rainfall. It is commonly found in central and peninsular India, mainly in the forested regions of Madhya Pradesh, Chhattisgarh, Maharashtra, Andhra Pradesh, some areas of Uttar Pradesh, and sub-Himalayan tracts, up to 1000 m altitude [20]. 

The species performs best in fertile, deep, clayey loam soil with good drainage and can tolerate extreme temperatures during the summer season. Wild populations have rapidly disappeared, and even single young saplings are rarely found in the forest. The International Union for the Conservation of Nature (IUCN) evaluated *P. marsupium* as a vulnerable species based on autogenic reproductive deficiency [10]. In addition, this species has been categorized by several researchers or conservation agencies as vulnerable [23], depleted [24], fast disappearing [25], endangered [26], critically endangered in Nepal [27], and vulnerable in Sri Lanka [28]. *P. marsupium* can be identified in the wild by its upright bole, longitudinally fissured bark, imparipinnate leaf arrangement (5–7 leaflets, 8–13 cm long), coriaceous, dark green and shiny leaves, scented yellow flowers in large panicles (1–5 cm long), and orbicular and winged fruits with flat pods. Each pod contains one to three seeds, bony and convex in shape. Flowering begins in November and continues up to March. The mature tree attains a height of up to 30 m and a girth up to 2.5 m, with clear and straight bole [29].

## 3. Phytochemistry and Therapeutic Values of *P. marsupium*

### 3.1. Active Constituents

**Figure 2:**
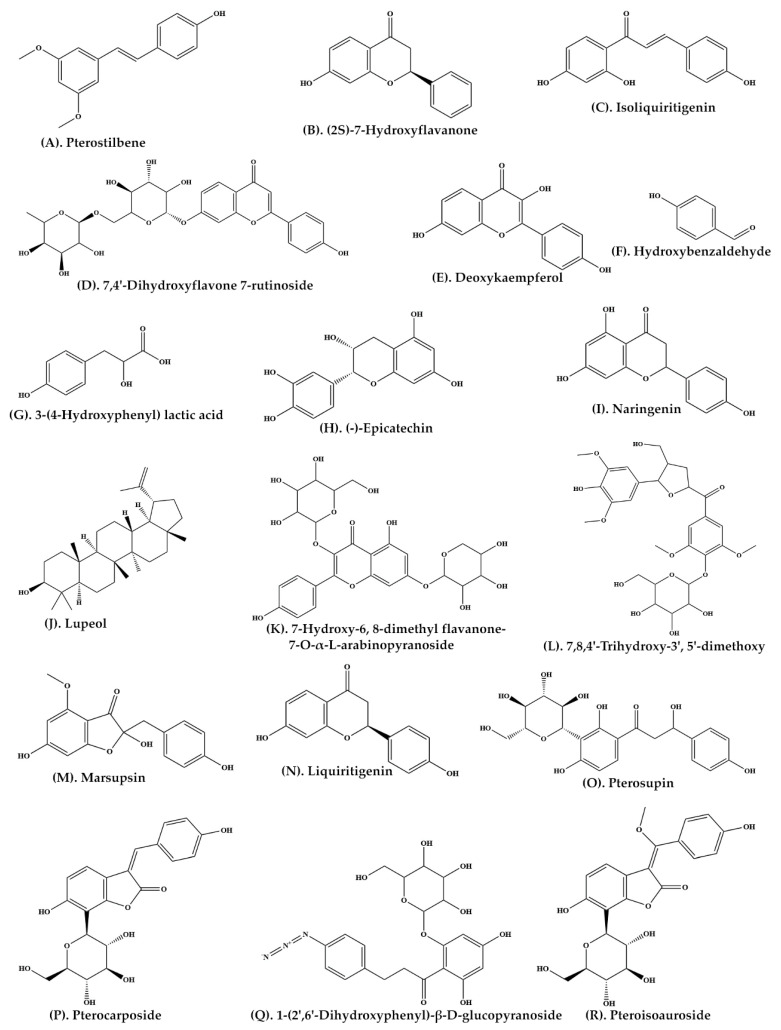
Molecular structure of bioactive compounds extracted from *Pterocarpus marsupium* Roxb.

**Figure 3:**
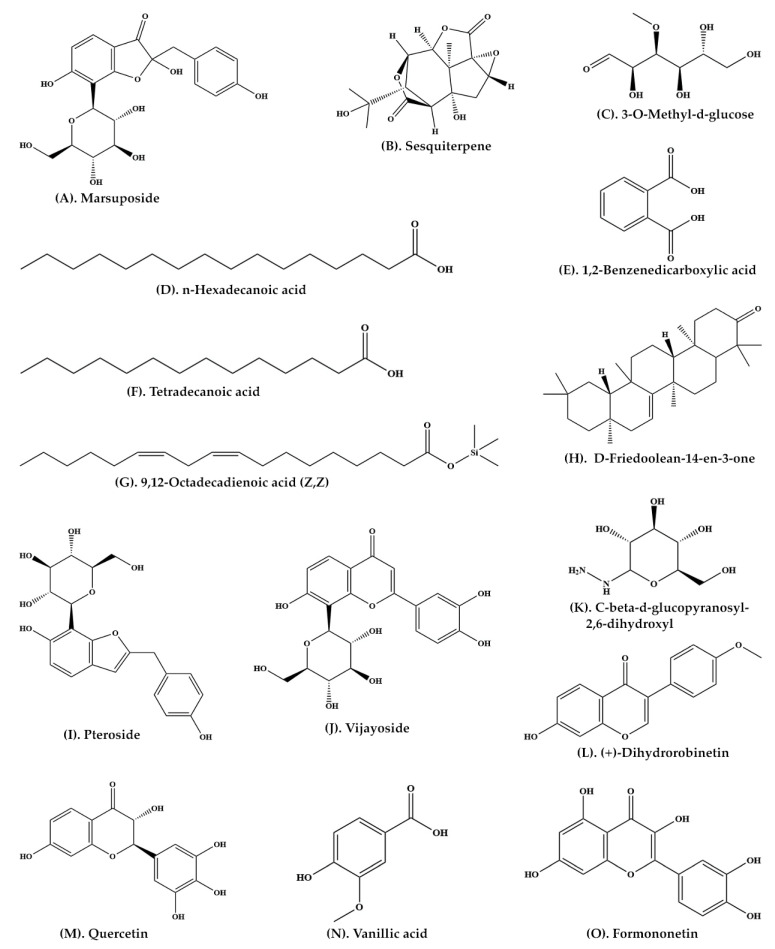
Molecular structure of bioactive compounds extracted from *Pterocarpus marsupium* Roxb.

A large number of important phytochemicals, such as glucosides, sesquiterpene and vijayoside (Table 1) have been isolated from aqueous extract of heartwood of *P. marsupium* [30]. The extract of heartwood contains pterostilbene (Figure 2A, pterosupin) (Figure 2O, marsupsin (Figure 2M), and liquiritigenin (Figure 2N), (−)-epicatechin) (Figure 2H) [31,32]. Bark extract contains several reputed phytochemicals such as 3-*o*-methyl-D-glucose (Figure 3C), n-hexadecanoic acid (Figure 3D), 1,2-benzenedicarboxylic acid (Figure 3E), tetradecanoic acid, (Figure 3F), 9,12-octadecadienoic acid (Z,Z) (Figure 3G), D-friedoolean-14-en-3-one (Figure 3H), and lupeol (Figure 2J) [33]. At least eleven bioactive compounds, namely pterocarposide (Figure 2P), 2,6-dihydroxyphenyl glucopyranoside (Figure 2Q), pteroside (Figure 3I), vijayoside (Figure 3J), pterosupol, marsuposide, epicatechin, quercetin (Figure 3M), vanillic acid (Figure 3N), formononetin (Figure 3O), and naringenin have been extracted from the heartwood of *P. marsupium* [21]. The most important bioactive compounds extracted from *P. marsupium* are presented in Table 1. Structures of bioactive compounds of *P. marsupium* are shown in Figure 2 and Figure 3.

### 3.2. Medicinal Properties

Ethno-medicine: Given the substantial evidence of its pharmacological properties, *P. marsupium* has potential as an herbal drug yielding tree; indeed, it has been used to cure several diseases in the Indian traditional medicine system for many centuries [30,45]. The flowers of the tree are used in the treatment of fever, and the heartwood powder is useful in treating chest pain, body pain, and indigestion [46]. Trivedi [47] reported that a paste made from wood and seeds is useful in treating diabetic anemia. In addition, Yesodharan and Sujana [48] have suggested that heartwood is useful in the treatment of body pain and diabetes. Interestingly, a cup made of the wood of *P. marsupium* heartwood is used for drinking water to control blood sugar levels in “Ayurvedic” medicine [49]. Aqueous infusions of the bark have also been used to treat diabetic patients since ancient times [50]. The stem bark is used to treat urinary discharge and piles, and the resin-gum is applied externally in the treatment of leucorrhoea [51]. The medicinal values of the active constituents or aqueous extracts of *P. marsupium* are shown in Table 2.

Anti-diabetic properties: Heartwood extract of *P. marsupium* was primarily used in the Ayurvedic system of medicine for treating diabetic patients and has been compared to proprietary drugs often prescribed to treat diabetes, such as metformin, which is mainly an anti-hyperglycemic medicine [64,65]. In earlier studies, an extract of *P. marsupium* bark was found to contain (−)-epicatechin, an anti-diabetic compound that promotes regeneration of pancreatic β-cells [66]. Subsequently, Manickam, Ramanathan, Farboodniay Jahromi, Chansouria, and Ray [32] reported two of the most important phenolic constituents of the heartwood, namely marsupsin and pterostilbene, which significantly reduced the blood glucose level in hyperglycemic rats. In yet another study [67], both of these compounds (marsupsin and pterostilbene) were shown to have insulin-like activities similar to those of metformin (1,1-dimethylbiguanide), an important hypoglycemic compound that stimulates glycolysis and inhibits glucose absorption in the intestine. These results indicate that both pterostilbene and marsupsin are powerful anti-diabetic agents that might be useful in the treatment of non-insulin-dependent diabetes mellitus patients.

Suppression of platelet aggregation: The oral administration of an extract from the leaves or stem bark of *P. marsupium* has been shown to inhibit the reduction of platelets, which suggests action against platelet aggregation. The heartwood extract, containing pterostilbene, also exhibited suppressive effects on platelet aggregation [52]. Another study [68] suggested that pterostilbene is a derivative of resveratrol—commonly known as one of the potent inhibitors of platelet aggregation. In a comparative study of pterostilbene and resveratrol [61] it was found that 50 µM pterostilbene resulted in 91% inhibition of platelet aggregation in rats, compared with 84% reduction after an equimolar dose of resveratrol. These results clearly indicate that pterostilbene is a potential substitute of resveratrol for platelet aggregation inhibition.

Dipeptidyl peptidase-4 (DPP-4) inhibition activity: Dipeptidyl peptidase-4 (DPP-4) is an intrinsic membrane protein dispersed in several tissues, including intestinal epithelial cells, renal proximal tubules, and the cells of the lungs, liver, kidney, and placenta [69]. Presently, there are three important DPP-4 inhibitors, namely Sitagliptin, Vidagliptin, and Saxagliptin, that are commercially available in the international market and used for the treatment of type-2 diabetes and Alzheimer’s disease (Type-2 diabetes is one of the major risk factors associated with Alzheimer’s disease). In several scientific studies, we note that it has been suggested that heartwood extract of *P. marsupium* has dipeptidyl pepdase-4 (DPP-4) inhibition properties in the treatment of type-2 diabetes and Alzheimer’s disease [59,70].

Cardiotonic activity: The aqueous extract of *P. marsupium* heartwood contains 5,7,2-4 tetrahydroxy isoflavone 6-6 glycoside, which is a potent antioxidant believed to prevent cardiovascular disease [45]. Digoxin, isolated from *Digitalis lanata*, is a most effective cardiotonic medicine but shows some acute side effects, such as gynaecomastia, irregular heartbeat, and poor kidney function [71]. Mohire, Salunkhe, Bhise, and Yadav [45] reported interesting results from a comparative study of the aqueous extract of *P. marsupium* heartwood and digoxin on the positive intropic and negative chronotropic effects on the heart (frog). They found that the heartwood extract showed a decrease in a heartbeat (negative chronotropic) and an increase in the height of force of concentration (positive intropic) effect. In comparison, a low concentration of digoxin increases the height of force of concentration (20%), which is four times less than the height of force of concentration produced by an aqueous extract of *P. marsupium* heartwood (80%). These results clearly suggest that the therapeutic efficacy of *P. marsupium* heartwood extract is much higher than that of digoxin. Thus, the limitations of digoxin can be overcome by using an aqueous extract of *P. marsupium* heartwood, which has been found to have excellent cardiotonic activity as well as a wide margin of safety compared with digoxin.

Lipid-lowering capacity: The condition of obesity is characterized by the deposition of extra fat along with high levels of fatty acids, glycerol, and pro-inflammatory markers, which can indicate a diabetic phenotype [72]. A continuous increase in body mass and weight gain increases the likelihood of type-1 and -2 diabetes [73]. In a recent experiment, Singh, Bajpai, Gupta, Gaikwad, Maurya, and Kumar [21] demonstrated that heartwood extract of *P. marsupium* potentially reduced the extra fat accumulation in adipocytes cells and was also conducive to the successful treatment of diabetic patients.

Cyclooxygenase (COX-2) inhibition and anti-inflammatory activities: Cyclooxygenase (COX) plays an important role in body tissue inflammation and inflammatory pain. It is the rate-limiting enzyme in the conversion of arachidonic acid to prostaglandin. A survey of the literature shows that there are principally three isoforms of COX that have been identified: COX-1 [74], COX-2 [75], and COX-3 [76]. Among these, COX-2 plays an important role during inflammation since it is the main agent to induce prostaglandin production during inflammation. In a study by Hougee, Faber, Sanders, de Jong, van den Berg, Garssen, Hoijer, and Smit [52], it was reported that pterostilbene—the active compound found in heartwood extract of *P marsupium*—shows selective COX-2 inhibitory activity in humans. In addition, pterostilbene is a stimulating compound used in the treatment of several inflammatory diseases [77].

Anticancer, anti-proliferative, analgesic, and antioxidant activities: Pterostilbene is a naturally occurring stilbenoid phytochemical synthesized in plants via the phenylpropanoid pathway [78]. Research has shown that it is a structural analog of various highly bioactive compounds such as stilbene and resveratrol [60,79]. Because pterostilbene (*trans*-3,5-dimethoxy-4′-hydroxystilbene) has a close structural similarity with resveratrol (3,5,4′-trihydroxy-trans-stilbene), it yields a large number of health benefits (Table 2). A number of researchers believe that pterostilbene has more potential bioactivities (i.e., anticancer, antioxidant, anti-inflammatory) than resveratrol [54,80,81]. In addition, Ferrer, et al. [82], Tolomeo, Grimaudo, Cristina, Roberti, Pizzirani, Meli, Dusonchet, Gebbia, Abbadessa, Crosta, Barucchello, Grisolia, Invidiata and Simoni [53], and Chakraborty, Gupta, Ghosh and Roy [55] reported that pterostilbene did not show any cytotoxic effect against normal human cells and inhibited certain types of cancerous cells.

## 4. Propagation of *Pterocarpus marsupium*

The plant consists of various organs and tissues, which in turn are made up of different individual cells. Plants are propagated through various methods: direct seed culture, explant cultures (from plant cells, tissues, organs, mature or immature zygotic embryo) on artificial media under aseptic conditions. Micropropagation is one of the best methods for obtaining genetically identical clones of donor plants [13]. This method has been extensively utilized for regeneration and conservation of various tree species, including *Melia azedarach* [83], *Acacia ehrenbergiana* [84], *Albizia lebbeck* [85], *Lagerstroemia speciosa* [86], *Cassia alata* [87], and *Erythriana variegata* [88]. Such propagation methods make possible the mass multiplication of new cultivars of valuable medicinal woody trees that would otherwise take several years to develop via conventional methods. In the following sections, the important methods adopted for the propagation of *P. marsupium* are discussed concerning the existing literature.

### 4.1. Mechanism of Seed Germination

Seed germination, the most common method for multiplication in flowering plants, is a process in which physio-morphological changes result in the activation of the embryo. As the seed absorbs water before germination, the seed embryo elongates. Shu, et al. [89] have proposed that once the radicle has grown out from the seed layers, the process of seed germination is complete. The processes involved in seed germination and how they are affected by different physio-morphological barriers have been well documented in a wide range of plant families, including Fabaceae [90,91]. The moisture content of seeds and the ambient storage temperature are key factors in promoting seed viability. Generally, seeds of many plants fail to germinate (due to a number of factors) and subsequently pass through a phase of dormancy that delays the whole life cycle of the plant. Different treatments have been found to improve germination and seedling growth. Furthermore, the promotive role of plant growth regulators on the breaking of seed dormancy and germination has been studied from time to time in many plant species. The general mechanism of seed germination and different factors affecting the germination rate are discussed below.

Seed germination and formation of the seedling: The process of seed germination—the resumption of growth of the embryo, leading to the formation of a seedling—is supported by environmental conditions that favor vegetative development, such as suitable temperature, required level of humidity, and the availability of oxygen and inhibitory substance, if any.

Seed development: Embryogenesis can be divided into three phases of approximately equal duration. The first phase begins with the zygote undergoing embryogenesis concomitant with endosperm proliferation. This is basically a phase of cell division and cell differentiation. The second phase, the accumulation of storage compounds to support the process, marks the end of cell division. In the third or final phase, the embryo becomes tolerant to desiccation. In this process, a seed dehydrates, losing up to 90% of its water. As a consequence of dehydration, metabolism comes to a stop and the seed enters a quiescent stage as a mature seed.

Hormonal balance of seed development: Abscisic acid (ABA) is the principal hormone involved in maturation and dormancy of an embryo. The content of ABA is very low during early embryogenesis, peaks (reaches maturation) during mid-embryogenesis, and declines when the seed attains maturity. However, some other hormones are also induced in the overall response of seed development. An example is the peak of ABA production in seeds, which coincides with a decline in the level of IAA and gibberellins [89,92]. Thus, an interaction between two or more hormones is a recurring theme in seed development in general. One difficulty in interpreting hormonal balance in seeds is that not all tissues in the seed are of the same genotype [93]. In a seed, the embryo and endosperm are the products of the female and male genotype products, whereas the seed coat is the exclusive product of the genome.

Seed dormancy: Dormancy of the seed, an adaptive feature, is a temporal delay in the process of germination and thus allows for (a) time for seed dispersal, (b) delay of germination until the onset of favorable conditions, and (c) maximization of seedling survival. If the environmental conditions are not suitable for germination, the seeds released in a dormant state remain in a dormant state. Embryo-imposed dormancy is intrinsic to the embryo. It is not affected by the seed coat or other surrounding tissues. The embryo dormancy is primarily due to the presence of an inhibitor such as ABA and the absence of a promoter such as gibberellin [89]. Finkelstein, et al. [94] reported that dormant seeds can be made to germinate subject to certain environmental conditions. Many species, such as tropical tree legumes, produce seeds with a tough seed coat, which protects against germination by restricting uptake of water and exchange of O_2_. These limitations can be removed by scarification. (In nature, abrasion by sand, microbial action, or passage through an animal’s gut have the same effect.) In some cases, the seeds are treated with mineral acid. Many seeds lose their dormancy while lying exposed on the ground during storage. During seed germination, an activated embryo requires the food reserves of cotyledons in dicot seeds and the endosperm in monocot seeds [95]. Food reserves, such as starch and proteins, are broken down by a variety of hydrolytic enzymes into sugars and amino acids.

### 4.2. Propagation from Seed

**Figure 4:**
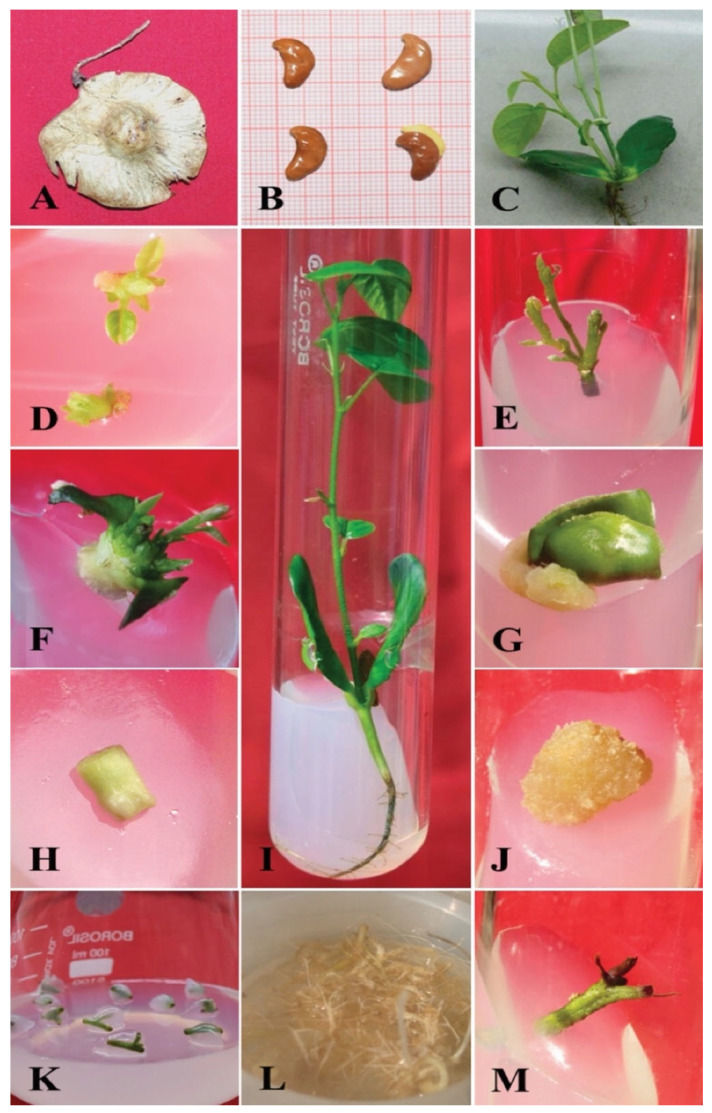
Different sources of explants (or planting materials) used for in vitro propagation of *Pterocarpus marsupium* Roxb. (**A**) Winged fruit, (**B**) 24 h presoaked seeds, (**C**) Intact seedling, (**D**) Shoot tip culture, (**E**) Juvenile nodal segment culture, (**F**) Cotyledonary node culture, (**G**) Cotyledonary leaf culture, (**H**) Hypocotyl segment culture, (**I**) Axenic seedling culture, (**J**) Callus culture via hypocotyl segment, (**K**) Synthetic seed culture, (**L**) Root culture, (**M**) Mature nodal segment culture.

The fruits of *P. marsupium* are orbicular winged pods (samara) and are the only planting material used (Figure 4A). The germination rate is very low (less than 30%) in natural conditions [96], probably due to the seeds being enclosed within a hard fruit coat and stony pericarp, resulting in what can be called mechanical dormancy [97]. The mechanical excision of the hard fruit coat is a tedious and labor-intensive task. Sometimes, pathogenic infection also restricts the natural propagation rate of *P. marsupium* [19]. Therefore, to overcome these constraints and improve the seed germination rate, several methods have been suggested, including seed viability test, wet-heat treatment, physical and acid scarification of seeds, excision of fruit coat by sharp scissors, and using different culture media compositions and seed orientations [19,20,98]. 

In the process of seed embryo growth, combinations of several cellular and metabolic pathways are coordinated by a complex regulatory network that regulates seed dormancy [89]. This period of growth is crucial to plant survival, given that the seed must germinate only when conditions—temperature, light, gases, water, seed coats, mechanical restrictions, hormonal structure, and level—are favorable. However, several plants show adaptive traits that promote survival under stressful conditions [94]. Many wild species that have been domesticated show a low level of seed dormancy compared to wild relatives, which ensures higher emergence rates after sowing [99,100]. However, the loss of seed dormancy is undesirable when it results in rapid germination of newly matured seeds and consequently extensive losses in quality and quantity of the crop, which presents problems for pre-harvest management, as well as industrial consumption [101].

In the case of ex vitro germination, any mechanism that will improve the absorbency of fruit coat could result in an improved percentage of seed germination. Wet heat therapy and physical fruit scarifications weaken the enclosure structure, which improves imbibition and thus enhances the germination rate of the seed. Generally, all these approaches are used more commonly in many plants’ fruit and seed dormancy pretreatments. They help to remove the many inhibitors partially and also to decrease the enclosure structure of fruits [102]. Sometimes, seeds’ long exposure to hot water may affect the embryo and reduce germination. The contrasting effects of hot wet therapy on the germination of *P. marsupium* and *P. santalinus* were stated by Kalimuthu and Lakshmanan [97]. It was observed that 24 h presoaked seeds (Figure 4B) showed decreased seed germination. Physically scarified seeds have been shown to be more successful in many cases relative to non-scarified and wet heat-treated seeds. Many physical limitations, such as gasses and the diffusion of moisture, are regulated by the thick seed coat of several plants, particularly the Fabaceae family. The high degree of impermeability is largely due to the presence of palisade coating in the seed coat of some legumes [103,104]. The dormancy seed, therefore, induces significant uncertainty in the germination of seed. Physical scarification of seeds has demonstrated that the increased germination rate is possibly attributed to the diffusion of water and gases, mainly by fissures, which causes various biochemical reactions after the rejuvenation of the embryo into a seedling [105,106]. In addition, improved germination patterns in chemically scarified seeds have also been documented relative to other treatments. Barmukh and Nikam [107] suggested that untreated seeds had a 28.2% germination rate within 15 days, whereas physically scarified seeds had a 55.3% germination rate. The treatment of H_2_SO_4_ for 30 min resulted in a maximum of 78–85% germination in ex vitro conditions. Treatment of scarification 30 min with concentrated H_2_SO_4_ can be enhanced, consistent and fast germination of seed for the development of *P. marsupium* seedlings. At this stage, the seedlings attained the height of 0.4 m after 8 months, and they were successfully acclimatized under field conditions. The removal of a chemical inhibitor and a scarification of H_2_SO_4_ seed coat is well explored in a variety of experimental studies on the seed germination of plants [108]. The practice of seed germination includes many important elements for the production of a mature plant under sufficient acid treatment and environmental conditions. The seed coat has an embryonic axis consisting of a radicle, plumule and cotyledons. Long exposure of fruit/seed to acid scarification was morphologically compromised embryonic axis, radicle, plumule, slow development of seedlings, cotyledonary leaf chlorosis, and leaf burnt on the margins [109,110]. All these characteristics are detrimental and decrease the food storage potential that sustains the embryo and its development until it has the ability to absorb sunlight and become autotrophic. In an earlier study, Kalimuthu and Lakshmanan [97] suggested that various combinations of sulphuric acid pretreatment had not changed in the germination of *P. santalinus* and *P. marsupium*, whereas in another study, *P. santalinus* fruit pods were pretreated with 1% H_2_SO_4_ for 4 days or concentrated H_2_SO_4_ for 5 min, the most effective treatment for seed germination was found [111]. In addition, Barmukh and Nikam [107] reported that the chemical scarification of seeds with concentrated H_2_SO_4_ for 30 min is the most effective treatment for homogenous and rapid germination of *P. marsupium*.

Several scientific studies have shown that early seed germination and seedling growth are tightly controlled by numerous hormonal signal molecules [90,112]. Different combinations of media, such as MS [113], B_5_ [114], and WH [115], were utilized for the assessment of in vitro seed germination in *P. marsupium* [116]; the highest seed germination rate (96%) was reported on half-strength-MS medium after 20 days. The orientation of the seed can sometimes be a significant factor in improving in vitro seed germination in many plant species [117]. In another study, Mishra, Rawat, Nema, and Shirin [19] reported that *P. marsupium* seeds cultured on a half-strength MS medium in a horizontal position showed maximum germination (78.23%). The results clearly showed that the seed orientation and strength of the medium both have a strong effect on in vitro seed germination (a significantly higher germination rate was obtained with horizontally cultured seeds compared with vertically cultured seeds). Similarly, the horizontal orientation of *Hardwickia binata* seeds significantly enhanced the germination rate compared with vertical orientation [118]. One more important aspect observed during in vitro germination practice is the optimization of an appropriate culture medium and the combination with plant hormones. Husain, Anis, and Shahzad [98] reported a maximum of 80% in vitro seed germination on half-strength MS medium enriched with 0.25 mg/L gibberellic acid, after six days of seed culture. This study clearly demonstrated that the combination of the appropriate media at the right strength along with the incorporation of plant growth regulator (GA_3_) enhanced the germination rate. Ahmad et al. [119] reported various allied parameters on in vitro seed germination, i.e., highest percent germination (91.3%), speed germination (29.0 seeds/day), coefficient germination (8.23%), mean germination time (12.15 days), and mean seedling height (10.31 cm) within 21 days of *P. marsupium* seed culture on 0.50 µM GA_3_ augmented MS medium. The enhanced concentration of GA_3_ promotes the rupture of seed testa and endosperm [120]. The use of culture media augmented with GA_3_ has been reported in many studies aimed at improving germination along with better elongation of the plantlets of different species [121,122,123]. It is also reported that GA3 regulated the synthesis of α-amylase in the aleuronic layer of seeds by upregulating the α-amylase gene, SLN1, and GAMYB transcription factors, thereby promoting germination [124]. Furthermore, DELLA mediated inhibition of BZR1 transcription factor has been shown to promote plantlet elongation [125]. GA_3_ stimulates the synthesis of mRNA, which is specific for α-amylase release and is assumed to be one of the factors for improving the rate of seed germination [126].

### 4.3. Micropropagation through Various Methods

In vitro propagation basically depends on the choice of appropriate explants (pieces of tissue used to initiate cultures) to serve as the preliminary experimental planting material. For multiple shoot bud induction (or bud breaking), the most frequently used explants are those that contain meristematic cells, such as cotyledonary nodes (CN), nodal segments (NS), immature zygotic embryo (IZE), hypocotyl segment (HS), shoot tips and root tip explants. The different types of explants obtained from 7-day-old axenic seedlings of *P. marsupium* are shown in Figure 4C–M. The cell division potential is highest in these tissues, which apparently yield the much-needed growth-regulating substances, such as cytokinins and auxins [127]. In vitro propagation highlights the potential of morphogenic responses on various explants of *P. marsupium* under the regime of different plant hormone combinations. However, the morphogenic potential of explants of various organs varies and some do not grow at all. Explants derived from juvenile seedlings are frequently used for organogenesis under the regime of different plant growth regulators, as they are easily established in axenic culture and have a greater morphogenic potential than do mature explants obtained from donor mother plants [128,129,130,131]. The axenic seedlings of *P. marsupium* are a suitable source for obtaining axenic planting material (or explants) as they are aseptically grown from sterilized seeds. Multiple shoot bud induction during plant cell, tissue, and organ culture greatly depend on the type of plant growth regulators (PGR) applied, and their concentration, uptake, transport, and metabolism, and the endogenous hormone levels of explants [132,133]. Endogenous levels of cytokinins in explants are available in various forms, such as free bases, nucleotides, ribosides, *O*-glucosides, and *N*-glucosides [134]. Exogenously supplemented PGRs can modulate the action of enzymes that control the level of endogenous hormones and enzymes [135]. In the next section, we will cover the progress made to date in in vitro propagation of *P. marsupium* through various explants under PGR regimes.

#### 4.3.1. Cotyledonary Node Culture

Cotyledonary node (CN) explants obtained from axenic seedlings of *P. marsupium* have been shown to have the highest regeneration frequency among explants [22,25,136,137]. Similarly, CN explants have been reported to show significantly greater morphogenic potential than do other explants in many tree species, including *Dalbergia sissoo* [138], *Acacia ehrenbergiana* [84], *Eucalyptus saligna* [139], *Lawsonia inermis* [140], *Prosopis cineraria* [141], and *Aegle marmelos* [142]. A variety of cytokinins, i.e., *meta*-topolin (*m*T), 6-benzyl adenine (BA), Kinetin (Kn), 2-isopentenyladenine (2iP), and thidiazuron (TDZ) has been used for *P. marsupium* micropropagation. In Table 3, we have summarized the in vitro propagation successes in this species.

**Figure 5:**
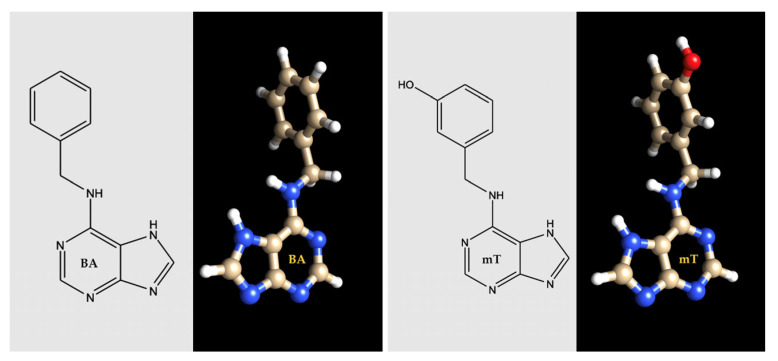
Comparative molecular structure of 6-benzyladenine (BA) and *meta*-topolin (*m*T).

Ahmad and Anis [22] reported that the highest number of shoots was obtained in CN explants when cultured on 7.5 μM *m*T containing MS medium, which is better than BA for shoot multiplication in *P. marsupium*. The superiority of *m*T over other cytokinins for in vitro propagation has been well documented in many species [151,152,153,154,155]. *Meta*-topolin, a benzyladenine analog [*N* 6-(3-hydroxybenzylamino) purine] is a highly active aromatic cytokinin [156] and differs from BA by having a hydroxyl group in the aromatic side chain (Figure 5), which facilitates the formation of a *O*-glycoside [155] capable of rapid conversion to the active form of nucleosides, nucleotides, or free bases when required [152]. Furthermore, these bases can be converted by *N^6^*- and *N^9^*-glycosylation or alanine conjugates of the purine ring into biologically inactive forms, i.e., stable derivatives [157,158,159]. Although BA is a widely used cytokinin in micropropagation systems, it has sometimes inhibited rooting efficiency, toxicity, and abnormal growths such as basal callus formation in many species [154,156,160,161]. The undesirable properties of BA may be due to its *N^7^*- and *N^9^*-glycosylation or conjugation with alanine that results in biological inactivation by the formation of chemically stable derivatives [156,162]. Therefore, utilization of *m*T in plant tissue culture has gained increasing interest due to reports of various important parameters, such as improved rate (%) of seed germination [163,164], enhanced multiple shoot induction [165,166], increased shoot length and quality [167,168,169], successful rooting [170,171], better quality of regenerated plantlets [164,172], easy acclimatization [171,173], alleviation of hyper-hydricity [171,174], delayed senescence [175,176,177], improved histogenic stability [178,179], alleviated shoot tip necrosis [179], improved physiological and biochemical activities [156,158,176], increased yield [180], and improved biomass content [22,156,173].

**Figure 6:**
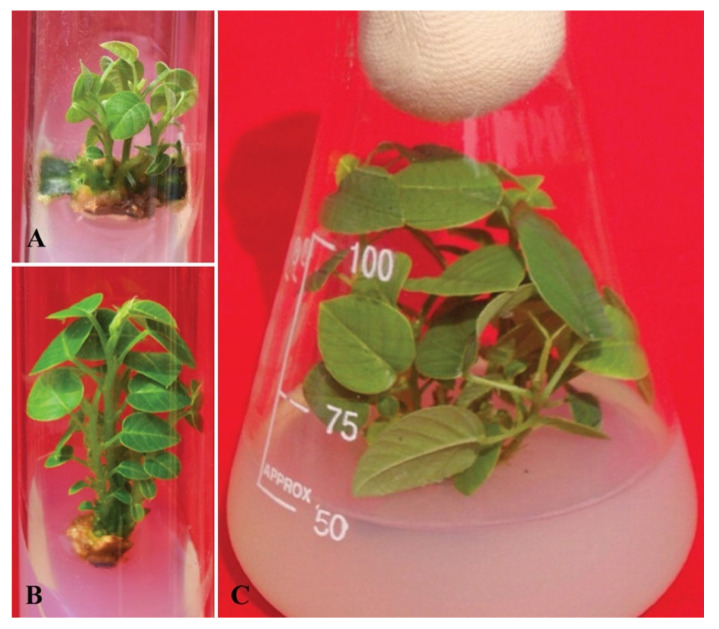
In vitro shoot morphogenesis in *Pterocarpus marsupium* Roxb. (**A**) Multiple shoots on MS + 0.5 μM TDZ, after 3 weeks. (**B**) Multiplication on MS + 5.0 μM *m*T, after 6 weeks. (**C**) Multiple shoots proliferation on MS + 0.5 μM GA_3_ + 0.5 μM TDZ + 1.0 μM NAA, after 9 weeks.

Interestingly, while Husain, Anis, and Shahzad [137] observed several shoots from CN explants when cultured on MS medium containing only 0.4 μM TDZ (Thidiazuron), yet the proliferation and shoot elongation remained undifferentiated for one months before these TDZ-exposed cultures were moved to the 5.0 μM BA fortified medium, which in turn induced the proliferation and subsequent shoot elongation. Studies specifically suggested that constant TDZ exposure inhibited shoot elongation [181]. In order to counteract such an antagonistic influence of TDZ, TDZ-exposed cultures have been moved to a secondary medium either free of hormones or supplemented by another PGR such as BA for development and elongation. Thidiazuron (N-phenyl N′-1,2,3-thiazol-5-yl urea) is a synthetic phenylurea having a potent cytokinin-like activity [182]. TDZ is widely used as an alternative to cytokinin in the plant tissue culture system (Figure 6A–C). It is a more powerful plant growth regulator (PGR) relative to other cytokinins for the induction of multiple de novo shoots in many plant species [88,183,184,185]. Murthy et al. [186] reported that exogenous usage of TDZ in culture media increased the endogenous amount of plant hormones, especially auxins and cytokinins, in micropropagation practices. TDZ also influences the cytokinins biosynthetic or metabolic processes of cytokinins responsible for controlling the concentration of endogenous levels of purine metabolites [187]. Several researchers concluded that augmentation of TDZ in culture media showed a favorable response on axillary shoot bud induction or bud breaks. However, in many plant species such as *Rhododendron* [188], *Adhathoda beddomei* [189], *Dalbergia sissoo* [138], and *Rauvolfia tetraphylla* [190,191], these shoot buds have not been elongated and proliferated. Many research studies have strongly indicated that such a detrimental impact of TDZ-exposed crops could be due to the existence of phenyl groups and have demonstrated many drawbacks, including bunching of microshoot, fasciation, hyperhydricity, low microshoot efficiency, and inhibition of rooting ability [192,193,194,195]. Another study by Huetteman and Preece [196] addressed the issue of shoot elongation by following a two-step culture strategy consisting of a primary medium fortified with TDZ for the induction of multiple shoot buds, followed by their transfer to a secondary medium fortified with another plant hormone (i.e., BA) that promotes shoot elongation. Such a type of culture strategy of using primary medium (shoot bud breaking/induction medium) and secondary medium (shoot elongation medium) has been successfully applied to a several tree species [137,147,197,198,199].

Anis, Husain, and Shahzad [25] examined the responses of CN explants of *P. marsupium* to optimize the maximum shoot multiplication under BA and Kn regimen alone or in combination with auxins and obtained good response when cultured on MS medium fortified with 5 µM BA which produced 7.83 shoots per CN explant within 6 weeks of culture. Whereas Kn induced a single shoot, and no significant response was detected. Meanwhile, the explants cultured on the BA fortified medium showed significant shoot growth, and were considered to be more responsive than Kn. The superiority of BA over Kn for multiple shoot induction from CN explant has been reported earlier in *Pterocarpus* species [136,200,201]. BA and Kn cytokinins are commonly used in plant tissue culture systems. However, the efficacy of BA over Kn in in vitro morphogenesis has been well documented in several plant species, such as *Curcuma zedoaria* [202], *Aegle marmelos* [203], *Andrographis paniculata* [204], *Albizia lebbeck* [205], *Terminalia bellirica* [206], *Acacia ehrenbergiana* [84], and *Acacia gerrardii* [207]. In addition, Kn has also been shown to be efficient in successfully developing in vitro propagation protocols for several species [208,209,210,211].

Chand and Singh [136] have developed a protocol for in vitro plant regeneration through CN of *P. marsupium*. An average of 9.5 shoots per CN explant were induced on MS medium containing 4.44 µM BA and 0.26 µM NAA in 85% cultures after 15 weeks. According to Chand and Singh, BA was more effective than Kn for multiple shoot formation from the CN explant of *P. marsupium*. It has been found that auxins could not induce shoot multiplication when used alone and that the cytokinin unaided produced very few shoots, whereas the combination of cytokinin and auxin underwent profusion of regeneration. Many studies have shown that a lower dose of auxins is needed in combination with cytokinin to boost the multiplication rate in many plant species [83,84,212,213,214]. α-naphthalene acetic acid (NAA), indole-3-acetic acid (IAA), and indole-3-butyric acid (IBA) are widely used in shoot multiplication, proliferation, and rooting cultures. Recently, the MS medium fortified with 7.5 µM *m*T and 1.0 µM NAA exhibited the highest frequency of shoot regeneration in *P. marsupium* [22]. Thus, the use of cytokinin to auxin in a ratio proven to be successful in high in vitro shoot regeneration instead of using cytokinin alone. The need for a low auxin concentration in combination with cytokinin can also regulate the endogenous cytokinin level by inhibiting the cytokinin biosynthesis gene, such as isopentenyl transferase (*PsIPT1* and *PsIPT2*) in micropropagated cultures [215,216]. Such type of synergistic effect of cytokinins (BA, Kn, or *m*T) with auxins (IAA, IBA, or NAA) on shoot multiplication and proliferation has been reported by several researchers in many plants’ species [217,218,219]. Cytokinin-auxin has also been associated with many physiological and developmental mechanisms, such as in vitro somatic embryo induction, apical dominance, cell cycle modulation, lateral root induction, control of senescence, and vascular tissues formation [135,220]. Tippani, Nanna, Mamidala, and Thammidala [148] also reported that the addition of auxin (NAA) with cytokinin (BA) to the medium increased the frequency of somatic embryo differentiation rather than BA individually in the medium. In that study, it was concluded that the MS medium containing 4.40 µM BA in combination with 2.69 µM NAA was the most suitable phytohormone combination for indirect organogenesis practice in *P. marsupium*. Several scientific studies have indicated that auxin controls the biosynthesis pathway of cytokinin through the unique activation of *IPT5* and *IPT7* genes [221,222]. In addition to these, several other studies have indicated that cytokinin and auxin jointly regulate their metabolism and signaling pathways [126,223]. As a result, both hormones were considered to be a key signaling molecules regulating the morphogenic response of plants.

#### 4.3.2. Nodal Segment Culture

A successful and efficient regeneration protocol from the nodal segment of *P. marsupium* was established by Ahmad, Ahmad, and Anis [147]. The NS is a good source of axillary shoot multiplication in the tissue culture system, and even a single explant can give rise to multiple copies of true-to-type plantlets within a few months. A two-fold culture strategy was applied to address the problem of shoot elongation. Primarily, the authors evaluated the effect of TDZ on nodal explant for axillary buds breaking on half-strength MS (liquid) medium containing 10 µM TDZ for 8 days. Thereafter, pretreated NS were transferred to a secondary medium containing different concentrations of *meta*-topolin. TDZ-pretreated NS—when transferred to MS medium supplemented with 5.0 µM *m*T in combination with 1.0 µM NAA—gave the best results, producing the highest number of shoots within eight weeks of culture. A similar culture strategy using shoot bud breaking (primary medium) and subsequent shoot elongation (secondary medium) has been effectively applied in many tree species, for example, *Acacia catechu* [224], *Eucalyptus grandis* [225], *Acacia sinuate* [198], *Lagerstroemia parviflora* [226], *Malus alba* [199], *Melia azedarach* [83], *Balanites aegyptiaca* [227], and *Acacia ehrenbergiana* [84].

Generally, in woody species, NS inoculated on hormone-free MS medium do not show any response, but shoot induction has been observed in cytokinin-augmented MS medium. Husain, Anis, and Shahzad [143] reported that the addition of 20 µM adenine sulfate (AdS) in MS medium containing 4.0 µM BA and 0.5 µM IAA improved the establishment of nodal explant cultures. A nucleotide base of adenine in the form of AdS can induce cell growth and significantly promote multiple shoot growth. AdS provide an additional nitrogen source to the cell, and this form of nitrogen can generally be taken up more rapidly than inorganic nitrogen. In a number of studies, the application of AdS in culture medium is recommended because it promotes the regeneration capacity of explants, especially in woody species such as *Tectona grandis* [228], *Bauhinia vahlii* [229], and *Melia azedarach* [230]. However, in the place of AdS, some other adjuvants, such as silver nitrate (AgNO_3_), casein hydrolysate (CH), polyvinylpyrrolidone (PVP), activated charcoals, coconut water, amino acids, and vitamins have been used in the culture medium for inducing shoot/root organogenesis and somatic embryogenesis [231]. Fuentes et al. [232] found that the addition of AgNO_3_ and fructose promoted somatic embryogenesis in *Coffea canephora*, whereas CH in combination with ammonium chloride has been shown to be the best combination in culture media to promote embryo germination as well as plant regeneration in *Sapindus mukorossi* [233].

#### 4.3.3. Shoot Tip Culture

Now-a-days, shoot tip culture techniques have been attracting interest by plant tissue culturists mainly due to their possible usage in large areas such as clonal multiplication of vegetatively propagated crop plants, virus elimination, genetic modification, germplasm conservation, etc. The most desirable aspect of shoot tip culture is that it has an advantage over other in vitro processes, the ability to preserve a high degree of genetic integrity among regenerants. Nehra and Kartha [234] have thoroughly reviewed the different facets of shoot tip culture and its commercial applications in many fields. Many researchers have used shoot tip explants to establish an effective in vitro regeneration protocol in several woody species. Sharma et al. [235] induced multiple shoots of *Bougainvillea glabra* from shoot tip explants culture on medium supplemented with of 0.5 mg/L BAP and 1.5 mg/L IAA. Multiple shoots were successfully achieved in shoot tip explant of *Catha edulis* (mature tree) when cultured on MS medium supplemented with 0.3 mg/L BA and 3.0 mg/L IAA by Hamid [236]. Kaur and Kant [237] reported a successful micropropagation protocol for *Acacia catechu* through shoot tip explants. It produced the highest number of shoots when cultured on MS medium containing 1.5 mg/L BAP plus 1.5 mg/L Kn. Shoot tip explants obtained from 20-day-old in vivo germinated seedling of *Pterocarpus santalinus* proliferated into multiple shoots on 1.0 mg/L BAP in combination with 0.1 mg/L fortified MS medium [238]. Similarly, Al-Sulaiman and Barakat [239] successfully induced multiple shoots of *Ziziphus spina-christi* from shoot tip on MS medium supplemented with 1.0 mg/L BA and 0.1 mg/L NAA. Recently, Hussain et al. [240] reported the highest differentiation of shoots *Tecoma stans* by culturing shoot tip on 7.5 µM BA and 0.5 µM NAA enriched MS medium. Their studies indicate that the formulation of PGRs in culture media for shoot tip culture depends mainly on plant species. Shoot tip culture is the most appropriate method for obtaining both virus-free and true-to-type clones. Up to now, only a single report has been published on successful in vitro regeneration of *P. marsupium* through shoot tip explants [149]. The highest shoot bud differentiation was achieved in this investigation at 7.0 µM *m*T plus 1.0 µM NAA, whereas Das and Chatterjee [96] attempted to regenerate multiple shoots from the shoot tip of *P. marsupium*. Initially, it was observed that shoot tip explants grew at 0.2 mg/L of BAP enriched MS medium, but after some time, it did not grow and eventually became necrotic on the same medium. As a result, these studies demonstrate that the formulation of culture media, PGRs and culture conditions plays an important role in effective shoot tip culture. 

#### 4.3.4. Immature Zygotic Embryos Culture

In plant tissue culture systems, the combination of PGRs plays an important role in determining the morphogenic response of explants. Mature tissues, which lack undifferentiated totipotent cells, respond poorly to regeneration treatments. Tippani, Vemunoori, Yarra, Nanna, Abbagani, and Thammidala [56] used immature zygotic embryos (IZEs) of a *P. marsupium* as the explant source, which, unlike other types of explants, is rich in meristematic cells. The researchers induced the highest frequency of shoot regeneration (93.8%) on MS medium fortified with 3.0 mg/L BA in combination with 0.5 mg/L IAA, which resulted in a maximum of 17.3 shoots per IZE explant. Subsequently, when these cultures were sub-cultured on MS medium containing a reduced concentration of 1.0 mg L^−1^ BA, an improvement in the average number of shoot (27.2) per IZE explant as well as increase in the average shoot length (4.5 cm) were documented. The potential use of IZEs to establish a plant regeneration system through shoot organogenesis and somatic embryogenesis has been verified in many tree species; for example, *Bixa orellana* [241], *Chamaecyparis obtusa* [242], *Pinus oocarpa* [243], and *Sapium sebiferum* [244]. IZEs are considered a reliable source of explants, and the combination of 2,4-D and Kn is generally recommended for somatic embryogenesis in several woody species angiosperms and gymnosperms [245].

In addition, the rate of multiplication of in vitro shoots depends greatly on the sub-culture of proliferating explants on a fresh medium. It is also imperative that as the number of cells, tissues, or organs becomes excessively high, there is a need to increase the amount of a culture or to increase the organ numbers for in vitro propagation. During extended cultures, the level of nutrients in the medium is significantly depleted, and as result of the development of certain harmful metabolites, a decline in relative humidity in culture vessels has resulted in the drying of developing cultures. Apóstolo et al. [246] advocated that the high humidity in culture flask or culture tubes helps to boost rapid growth. Thus, a repetitive sub-culture strategy can help in inducing multiple shoots, followed by improved rooting [217,247]. Several researchers have introduced a repeated sub-culture approach to improve multiplication rates in several plant species such as *Feronia limonia* [248], *Pterocarpus santalinus* [249], *Balanites aegyptiaca* [217], *Spondias mangifera* [250], *Arnebia hispidissima* [251], and *Tetrastigma hemsleyanum* [252].

#### 4.3.5. Intact Seedling Culture

Multiple shoot induction directly from the seed explant (also called intact seedling culture) is another method for rapid in vitro propagation (Figure 4C). The significant aspect of this regeneration protocol is that shoot differentiation occurs directly from the CN of the seedling, eliminating the need for an explant preparation step. Our literature review found that the intact seedling method greatly reduces many difficulties such as the cost of culture, time of regeneration protocol, and other considerations such as the age and size of the explant and its orientation in the culture vessel. However, it should be noted that these very same factors are thought to have an important role in successful regeneration, especially in leguminous tees. Malik and Saxena [253] reported that the rate of shoot multiplication via the intact seedling method in *Phaseolus vulgaris* was higher than that obtained from the explant method. Bhuyan et al. [254] developed a high throughput shoot multiplication protocol through intact seedling propagation of *Murraya koenigii* cultured on 5.0 mg/L BA and 0.4 mg/L GA_3_ in MS medium. The authors found that the application of GA_3_ to the medium had no stimulatory effect on shoot multiplication per intact seedling, whereas it did exhibit a remarkable effect on shoot elongation. Hussain, et al. [255] reported that the shoot multiplication rate from intact seedlings of *Sterculia urens* was highest when cultured on MS medium containing 5.0 µM BA, 0.4 µM GA_3_, and 0.1% ascorbic acid. Perveen, Anis, and Aref [85] successfully developed an in vitro regeneration protocol for *Albizia lebbeck* through the intact seedling method. In this trial, the highest shoot multiplication response was obtained from the highest dose of 5.0 µM TDZ (primary medium) followed by transfer to a lower dose (0.5 µM) of TDZ (secondary medium). Cumulatively, the results of these studies are clear evidence that intact seedling cultures are greatly influenced by the formulation of the growth regulator and that excision of the explant is not necessary for in vitro shoot multiplication. In case of *P. marsupium*, Ahmad, Anis, Khanam and Alatar [149] reported only direct shoot multiplication from the intact seedling method. The optimum culture, containing 0.50 µM GA_3_, induced the maximum seed germination response along with better shoot growth when compared to cultures with TDZ. Similarly, cultures treated with TDZ exhibited a higher shoot multiplication rate than did those treated with GA_3_. However, it was found that the two hormones combined together at an optimal level (TDZ at 0.50 µM and GA_3_ at 0.50 µM) resulted in the most effective treatment for germination response, shoot multiplication, and subsequent shoot elongation per explant. The authors [149] hypothesized that the improvement in protocol performance was due to the interaction of the two hormones, with successful regulation both in terms of biosynthesis and signal transduction.

#### 4.3.6. Somatic Embryogenesis

In somatic embryogenesis (SE), a single cell or group of somatic cells initiate the developmental pathway that leads to the formation of NZEs. This means that the embryos have no connection with pre-existing vascular tissue within the maternal callus. Williams and Maheswaran [256] suggested that somatic embryos can differentiate from pre-embryogenic determined cells, leading to direct embryogenesis. There are two well-defined methods for SE: the direct method (without callus) and the indirect method (with a callus-intervening phase). SE has been widely exploited in many plant breeding activities, such as mass-scale propagation, shortening of the breeding cycle, development of synthetic seeds, and genetic transformation of industrially important crops [127,231]. Currently, there are only a few published reports of SE in *P. marsupium* (Table 1). Differentiation of the somatic embryo can occur directly on the callus induction medium, whereas in many cases a combination of different media and hormones is required for callus induction, somatic embryo formation, and morphogenesis of shoot and plantlet conversion [144]. Recently, Tippani, Nanna, Mamidala, and Thammidala [148] developed an improved protocol for in vitro plantlet regeneration via SE from IZE explants of *P. marsupium*. Dedifferentiated tissues were induced profusely in 96.6% callus from IZE explants when cultured on a callus induction medium, i.e., MS plus 5.37 µM NAA. The callus was successfully induced to produce somatic embryos when cultured onto differentiation medium, i.e., MS consisting of 2.69 µM NAA and 4.40 µM BA. Lastly, maturation of somatic embryos into plantlets has been accomplished with the use of half-strength MS fortified with GA_3_ (5.80 µM). Chaturani et al. [257] reported that IZEs are an ideal explant to obtain the highest culture initiation and multiplication under in vitro conditions. There are many reports available on the utilization of IZE explants for inducing SE in tree species such as *Pinus oocarpa* [243] and *Fraxinus mandshurica* [258].

In addition, there have been many reports of the efficacy of HSs, obtained from aseptic seedlings, to achieve indirect SE in many plant species [259,260]. Husain, Anis, and Shahzad [144] achieved SE from the callus, derived from hypocotyl segment (excised from12-day-old axenic seedlings) of *P. marsupium*. Primarily, callus formation occurred on callus induction medium, i.e., MS containing 2,4-D (5.0 µM) and BA (1.0 µM). Secondarily, the calluses were successfully converted into somatic embryos when they were sub-cultured onto differentiation medium; i.e., MS containing 0.5 µM BA and 0.1 µM NAA in combination with 10 µM ABA. Husain, Anis, and Shahzad [144] also suggested that maturation of somatic embryos takes place by the addition of ABA, a growth inhibitor, which creates stress conditions that are conducive to the development and maturation of somatic embryos of *P. marsupium*. It may be possible that ABA interacts synergistically with both hormones (auxin-cytokinin) and stimulates maximum somatic embryo maturation. SE formation has occurred in many leguminous tree species via various explant sources such as immature cotyledon in *Acacia catechu* [261], immature cotyledon in *Hardwickia binata* [262], endosperms in *A. nilotica* [263], embryonic axes with cotyledons in *Albizzia julibrissin* [264], IZEs in *Acacia mangium* [265], and immature cotyledon in *Pterocarpus marsupium* [145].

### 4.4. Rooting and Acclimatization

**Figure 7:**
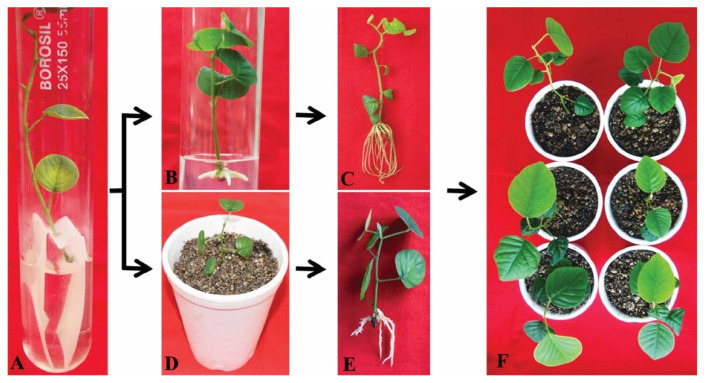
Two step rooting strategy for root formation in microshoots of *Pterocarpus marsupium* Roxb. (**A**) Pretreatment of microshoot in liquid medium employing filter paper bridge; (**B**) Pretreated microshoot transferred onto semi-solid medium containing phytagel for in vitro root formation; (**C**) 4-week-old in vitro rooted microshoot; (**D**) Pretreated microshoot transferred onto Soilrite (a synthetic soil) for ex vitro root formation; (**E**) 4-week-old ex vitro rooted microshoot; (**F**) Acclimatized plantlet in Soilrite.

Rhizogenesis of in vitro raised microshoots is often problematic in woody species and leads to significant economic consequences because of losses at this stage [266,267]. The development of a well-rooted system is vital for the successful establishment of in vitro raised microshoots in field conditions. Therefore, adventitious root formation is an important step for in vitro regeneration of various woody plants [268]. The use of auxins affects the in vitro root formation of microshoots raised in tissue culture [269]. Some important factors (type and concentration of auxin and treatment duration) play crucial roles in root induction [270,271]. IBA is an important exogenous auxin utilized for in vitro root induction in several plant species because it has shown more resistance to photodegradation, adherence to microshoots, and inactivation by biological action [272]. The stimulatory effect of IBA on induction of rooting may be due to successive rooting gene activation and better uptake, transportation, and stability compared with other auxins [159,273]. This type of two-step rooting strategy has been applied in many tree species [147,274]. In the first step, the distal ends of microshoots are dipped in a half-strength MS (liquid) medium containing a high dose of auxin (IBA) over a filter paper bridge for several days. In the second step, these pretreated microshoots are transferred onto half-strength MS (semisolid) medium, fortified with a lower dose of IBA, and cultured for one month (Figure 7A–F). Regenerated microshoots of *P. marsupium* have been affected by high dose IBA pretreatments, which improves the rate of rooting response, the number of roots per microshoot, and their subsequent length [25,56,143].

Ahmad and Anis [22] have reported that root forming efficiency in *m*T-derived microshoots is higher compared to BA-derived microshoots. Rhizogenesis in microshoots was carried out via a two-step rooting procedure. For the purpose, distal ends of microshoots were pre-treated with a high dose of IBA (100 µM) augmented ½ MS (liquid) medium for 5 days, followed by their transfer onto the half-strength of MS (semisolid) medium containing a low dose of IBA (1.0 µM) resulting in a successful root induction response. Ludwig-Müller [273] proposed that several factors, such as better absorption of auxin, transport, and stabilization over other auxins, and the subsequent genes, are responsible for the in vitro root formation. However, an inhibitory effect of BA on in vitro root development and low acclimation rates has also been recorded in many plant species [172,275]. On the other hand, a profound impact of *meta*-topolin on in vitro rooting and acclimatization of regenerated plantlets has also been identified in several species [161,173,276]. Exogenous doses of auxin in the culture medium are commonly used in different plants to induce in vitro rhizogenesis in regenerated microshoots [84,277,278]. Auxins serve as a central regulator of adventitious root formation. Specifically, it stimulates the preparation of pericycle cells that induce lateral roots [279,280]. De Smet [281] has reported several research studies on the use of auxin for the initiation and growth of lateral roots in isolated microshoots. The addition of exogenous auxin to the culture medium induces new primordial lateral root unrelated to acropetal branching in many plant species, including *Arabidopsis* [282]. In an experiment performed by Ilina, Kiryushkin, Semenova, Demchenko, Pawlowski and Demchenko [280], the main lateral root of the pericycle cells undergoes asymmetric anticline arises three adjacent pericycle cells. After some time, all active pericycle cells are arrested in the basal portion of the elongation region [283]. Since some of the pericycle cells that left the basal portion of the root apical meristem in the G1 phase of the cell cycle may begin the cell division of the cells involved in the induction of primordia’s lateral roots [284].

Jaiswal, Choudhary, Arya, and Kant [146] reported that being a woody perennial *P. marsupium* is difficult for in vitro rhizogenesis. In vitro root induction in microshoots found that IBA was more effective than the other auxin and ½ MS medium containing 4.92 µM IBA induced 2.14 roots per microshoots with an average root length of 1.24 cm in 42.2% cultures. On the other hand, in an experiment performed by Tippani, Vemunoori, Yarra, Nanna, Abbagani and Thammidala [56], the best response was recorded on MS medium containing 3.0 mg L-1 IBA pretreatment for 24 h, followed by transfer onto hormone-free MS medium, produced a maximum number of 3.2 roots per microshoot with mean root length of 2.9 cm in 70.8% cultures after 15 days of transfer. According to De Klerk et al. [285], high doses of auxin pretreatment are needed during the initial root induction process, but in many cases during root elongation, a high dose of any growth regulator becomes inhibitory. A large number of researchers have proposed IBA as the best rooting hormone in many plant species like *Kigelia pinnata* [286], *Terminalia arjuna* [287], *Albizia lebbeck* [85], and *Syzygium cumini* [214]. On the other hand, in *P. marsupium* microshoots, Husain, Anis, and Shahzad [137] applied a two-step rooting protocol for in vitro root induction. In the first step, the distal end of microshoots was pulse-treated with 200 µM IBA for 4 days and on filter paper bridge on ½ MS (liquid) medium, followed by step-two with subsequent transfer of these pulse-treated shoots to 0.6% agar-gelled ½ MS containing 0.2 µM IBA in combination with 3.96 µM phenolic acids (phloroglucinol). At this stage, the maximum number of 4.4 roots per microshoot with a mean root length of 4.0 cm was obtained in 65% of cultures after 4 weeks. In several tree species, the stimulatory effect of phloroglucinol has been documented [288,289,290]. Interestingly, De Klerk et al. [291] suggested that phloroglucinol in combination with auxin had a synergistic effect in inhibiting peroxidase activity in the culture, thereby protecting the endogenous auxin from peroxidase-catalyzed oxidation. Subsequently, an in vitro rooting response in microshoots of *P. marsupium* was also observed when it was grown on the primary liquid MS medium followed by switch to the secondary hormone-free semisolid half-strength MS medium. The highest root formatting frequency (70%) was achieved with a maximum 3.8 roots per microshoot on an average root length of 3.9 cm on the MS medium containing 100 µM IBA and 15.84 µM phloroglucinol after 4 weeks of culture [143]. Ahmad, Ahmad, Anis, Alatar, Abdel-Salam, Qahtan, and Faisal [150] recently reported an improvement in the in vitro rooting response in *P. marsupium* by pre-treating microshoot with 100 µM IBA for 5 days and then transferring to medium of containing 0.50 µM GA_3_. This combination has been shown to be the highest in vitro rooting in microshoots. Improvement in rooting response was hypothesized that IBA would be stored in basal ends of microshoots during pretreatment and would be actively used when interacted with GA_3_.

Recently, many tissue culturists have focused on ex vitro rhizogenesis in microshoots because ex vitro rooted plantlets produce a better developed root system compared with those of in vitro raised plantlets [129,292,293]. The ex-vitro rooting strategy is practical and cost-effective, requiring less time, less labor, fewer chemicals, and only minimal equipment compared with the requirements of in vitro rooting practice. Many researchers have reported that ex vitro rooted plantlets have good quality roots with minimum damage, are easy to acclimatize, and have a high survival rate because they do not require any additional acclimatization before being transplanted in natural conditions [85,129,294,295,296]. A major breakthrough in ex vitro rooting was achieved by Ahmad, Anis, Khanam, and Alatar [149] via a two-step rooting procedure. In this process, microshoots were isolated from in vitro grown cultures and subjected to the pretreatment of basal ends in optimized doses of IBA, followed by transfer onto various potting substrates. The best rooting response was obtained when the basal ends of microshoots were treated with 250 µM IBA for 5 days, followed by transfer to Soilrite, where a maximum of 3.63 roots per microshoot and mean root length of 3.59 cm, as well as the highest rooting frequency 67.7%, were recorded 4 weeks after transplantation. The authors reported that the ex-vitro rooted plantlets of *P. marsupium* were successfully acclimatized, with 96.7% survival rate [149]. Similarly, ex vitro rooting in regenerated microshoots has been achieved in a large number of woody species, including *Tectona grandis* [297], *Nyctanthes arbor-tristris* [298], *Vitex negundo* [183], *Malus zumi* [299], *Melia azedarach* [83], and *Tecomella undulata* [300]. In the study by Ahmad and Anis [22], the hardening of regenerated plantlets of *P. marsupium* was achieved by the well-rooted shootlets being gently removed from the culture vessel and washed with running tap water to remove any adherent; after which the plantlets were transplanted to Thermocol cups (10-cm diameter) containing sterile Soilrite. Other researchers have covered the culture cups with transparent polyethylene bags as a safeguard to ensure high humidity and placed the cups under 16/8 h (day/night) in culture room conditions [147,301]. These polybags were gradually opened in order to acclimatize the plantlets to natural conditions. Finally, the acclimatized plantlets were successfully shifted to normal garden soil under natural daylight conditions. Generally, tissue culture-raised plantlets have low photosynthetic rate because regenerated plantlets have underdeveloped photosynthetic apparatuses [302]. The improvement in photosynthetic pigment contents in regenerated plantlets is possible during acclimatization, as noted by several researchers [303,304]. The substantial increment in photosynthetic pigment content with high light intensity promotes the pigment biosynthesis enzyme, which is vital to synthesis of photosynthetic pigment content [305]. There have been several investigations of the abrupt decrease in photosynthetic pigment contents during the initial days followed by a continuous increase during the acclimatization processes in many plants [131,306,307].

## 5. Molecular Studies of *P. marsupium*

### 5.1. Genetic Fidelity Assay

Plant tissue culture techniques are an important tool in the clonal propagation of genetically uniform plants that possess desirable traits. Many researchers have suggested that the existence of somaclonal variations among sub-clones of an elite parental line is a potential drawback during micropropagation practice [240,308]. Javed, Alatar, Anis, and El-Sheikh [88] advocated that genetic uniformity of regenerated plantlets is a prerequisite to sustain the desired genotypes. The application of phytohormones at elevated concentrations and continuous sub-culturing of cultures for long periods hinders the maintenance of genetic stability [22]. The consequences of unintended mutations and the mixing of regenerated plantlets could mean that much time and money is wasted before the mistakes are discovered. Therefore, it is necessary to validate the uniformity of the regenerated plants at the genetic level through molecular markers, given that variability induced by continuous practice of in vitro propagation cannot be detected phenotypically because the structural differences in the gene products are not sufficient enough to alter the phenotypes [309]. Although many approaches have been attempted to detect genetic homogeneity (i.e., true-to-type clones) among regenerants, the most suitable technique appears to be DNA-based molecular markers. Such markers are not affected by environmental factors and can be analyzed using genomic DNA from any growth stage; therefore, these markers are very useful in analyzing genetic fidelity in a large number of plant species. RAPD and ISSR have become more popular among the different molecular marker techniques, as they do not require any prior DNA sequence information [87,191,310].

There have been scores of studies on genetic fidelity analysis by using RAPD and ISSR techniques in regenerated plants of different species such as *Moringa peregrina* [311], *Alhagi maurorum* [312], *Terminalia bellerica* [313], *Morus alba* [314], *Lawsonia inermis* [140], *Platanus orientalis* [315], *Erythrina variegata* [88], *Tecoma stans* [240]. Their simplicity, cost-effectiveness, and requirement of low quantity DNA are some reasons for the selection of these markers for genetic fidelity analysis [311,314,316,317,318].

Although DNA-based markers have been shown to be an effective technique to assess genetic fidelity in a number of plant species, there are few documented techniques particular to *P. marsupium*. In one recent study [149], assessment of genetic fidelity in *P. marsupium* regenerants was achieved using DNA-based ISSR primers. The authors of this study obtained a total of 35 bands, with an average of 4.38 bands per primer, in a monomorphic banding pattern. In an earlier study, Ahmad and Anis [22] had used a total of 29 RAPD primers to detect genetic fidelity among regenerated plantlets of *P. marsupium*; the results were monomorphic DNA bands exhibiting complete genetic uniformity. In still earlier work, Tippani, Vemunoori, Yarra, Nanna, Abbagani, and Thammidala [56] reported that four ISSR primers produced a monomorphic banding pattern regenerated when compared with mother plant. In a later experiment [148], eight ISSR primers were used for DNA amplification, producing a clear, reproducible, and monomorphic DNA banding pattern demonstrating genetic fidelity among regenerants of *P. marsupium* derived through the SE method. Taken together, this research confirms the efficacy of regeneration protocols for true-to-type plant breeding in general and *P. marsupium* in particular.

### 5.2. DNA Barcoding

Presently, several adulterants of *Pterocarpus* species are available in the international trade market [16]. Scientific authentication of genuine species is vital for the herbal drug industry; however, the conventional approach for the identification of authentic species is not sufficient. The difficulty lies in the fact that most of the physical and anatomical characteristics (i.e., wood density, grain, and color) of *Pterocarpus* species are very similar to its adulterants. DNA barcoding offers an alternative and feasible taxonomic tool for rapid and robust species identification in general [17,319,320,321] and could be an effective technique to distinguish *Pterocarpus* and its adulterants. Jiao, Yu, Wiedenhoeft, He, Li, Liu, Jiang, and Yin [16] developed a DNA barcode to distinguish adulterants of six commercially important species of *Pterocarpus* (*P. marsupium*, *P. santalinus*, *P. indicus*, *P. erinaceus*, *P. zenkeri*, *P. angolensis*). An array of genes, including *ITS2*, *matK*, *ndhF-rpl32*, and *rbcL*, have been utilized to discriminate *Pterocarpus* species through TaxonDNA and tree-based analytical methods. Jiao, Yu, Wiedenhoeft, He, Li, Liu, Jiang, and Yin [16] suggested that two of these genes, namely *matK* and *rbcL*, are the core DNA barcodes for authentication of many plant species. The *ITS2* regions are standard DNA barcodes, especially used in identifying adulterants of industrially important medicinal plants [9,322]. Similarly, *ndhF-rpl32*, an intergenic spacer gene of cpDNA, has the potential to be used for DNA barcoding of a large number of species [323,324,325]. Consequently, many researchers in this field have recommended DNA barcodes as well-known tools for authenticating genuine herbal raw materials in quality control programs and forensic studies [326,327,328].

### 5.3. Genetic Transformation

Establishment of in vitro propagation protocols is a prerequisite for genetic manipulation studies because in traditional breeding techniques—such as backcrossing and selfing—it is difficult to fix desirable alleles in a particular genetic background [13]. Successful transfer of genes of interest through genetic transformation technique has been well documented in many tree species [329,330,331]. Gorpenchenko et al. [332] established a successful genetic transformation process in *Panax ginseng* through callus-based shoot organogenesis. Similarly, Tippani, Yarra, Bulle, Porika, Abbagani, and Thammidala [145] demonstrated genetic transformation in *P. marsupium* through callus-based organogenesis using immature cotyledon explants obtained from 9-day-old axenic seedlings. Callus formation occurred on callus induction medium containing MS fortified with 1.07 µM NAA for 2 weeks. Subsequently, the callus was co-cultivated with *A. tumefaciens* (harboring the binary plasmid with *uidA* and *hpt* genes) and cultured on MS augmented with BAP (8.9 µM), NAA (1.07 µM), and acetosyringone (200 µM) for 2 days. These cultures were then transferred onto MS containing BAP (8.9 µM), NAA (1.07 µM), hygromycin (20 mg/L), and cefotaxime (250 mg/L) for multiple shoot induction, followed by transfer to shoot elongation MS medium containing BAP (4.40 µM), hygromycin (15 mg/L), and cefotaxime (200 mg/L). Putatively transformed microshoots were rooted on MS medium containing BA (2.85 µM) in combination with hygromycin (20 mg/L), after dip treatment of the distal ends of *P. marsupium* for 24 h in MS medium fortified with IBA (19.60 µM). Tippani, Yarra, Bulle, Porika, Abbagani, and Thammidala [145] validated the gene transfer into putatively regenerated plants through RT-PCR and GUS assay. Interestingly, Jube and Borthakur [333] proposed that the rate of transformation in woody species is meager because excised explants produce several harmful phenolic compounds. On a contrary note, there are many reports published on *A. tumefaciens*-mediated transformation in leguminous woody trees [330,331].

## 6. Conclusions and Future Prospects

The commercial productivity of tree plantations could be increased by reducing the genetic diversity of forest species and achieving greater homogeneity of tree phenotypes. Currently, many factors, such as increasing demand from pharmaceutical companies and timber-based industries and the overall decline of the global forest cover, as well as the impacts of climate change, have all motivated forestry decision-makers to raise the productivity of natural forests. There is also an increasing demand for aromatic and herbal drug yielding plants because natural products are seen to be non-toxic and have fewer side effects. Our comprehensive survey of the literature revealed that knowledge of the pharmacognosy, ethnobotany, and micropropagation of *P. marsupium*—a species of high value globally and in India—is rather limited and has only appeared in the past three decades. Plant biotechnology has opened new avenues for the generation of novel genetic variability, and techniques in this field now offer greater selection and are increasingly more precise and reproducible. Such techniques have broad applications in a number of important areas, for example, genetically modified food, feed, and fiber.

Micropropagation of *P. marsupium* can offer great advantages over traditional methods. Such advances can help researchers meet their goals in numerous specialties: plant breeding, plant biotechnology, germplasm conservation, rapid propagation of genetically modified plants, secondary metabolites biosynthesis, germplasm exchange, extensive collection within minimum space, supply of important planting material for wild population recovery, and molecular and ecological studies. Moreover, before developing any regeneration protocol, information on actual genetic variability and the cryptic number of the differentiated genetic resources are essential for both the genetic improvement of the species and its conservation. Before developing an effective method to maintain the genetic diversity of any targeted species, it must first be quantified. A promising method in regeneration programs is the use of DNA-based molecular markers.

Advances in DNA barcoding will help in the authentication of key forest species such as *P. marsupium* and may eventually lead to the formulation of legislation ensuring the public’s access to this plant at a reasonable cost. There are several substituted for *P. marsupium* wood on the market, and they can be less effective as medicines and, in some circumstances, fatal due to the toxicity of the substituted plant material as an open access, worldwide library of reference barcode sequences continues to be collected, DNA barcoding may be a viable answer to species authentication, allowing non-taxonomists to identify specimens. In vitro propagation and genetic diversity analysis of *P. marsupium* can be used effectively to select superior populations of the species for breeding programs aimed at improving productivity, wood quality, and chemical constituents, thereby helping to inform plans for conservation and sustainable use of this valuable plant species. More importantly, critical elements of an effective conservation strategies need to be discussed.

## Figures and Tables

**Table 1:** Important bioactive compounds extracted from *Pterocarpus marsupium* (in chronological order).

Plant Parts	Extract Preparation	Technique *	Bioactive Compound	References
Heartwood	Ethyl acetate	C-SG	Pterostilbene (Figure 2A) (2S)-7-Hydroxyflavanone (Figure 2B) Isoliquiritigenin (Figure 2C) 7,4′-Dihydroxyflavone 7-rutinoside (Figure 2D) 5-Deoxykaempferol (Figure 2E) *p*-Hydroxybenzaldehyde (Figure 2F) 3-(4-Hydroxyphenyl) lactic acid (Figure 2G)	[34]
Bark	Ethanolic extract	C-SG	(−)-Epicatechin (Figure 2H)	[35]
*P. marsupium* extract	Ethyl acetate	C-SG	Naringenin (Figure 2I) Lupeol (Figure 2J)	[36]
Roots	Ethanolic extract	C-SG	7-Hydroxy-6, 8-dimethyl flavanone-7-*O*-α-L-arabinopyranoside (Figure 2K) 7,8,4′-Trihydroxy-3′, 5′-dimethoxy flavanone-4′-*O*-β-D-glucopyranoside (Figure 2L)	[37]
Heartwood	Ethyl acetate	Thin Layer Chromatography	Marsupsin (Figure 2M) Liquiritigenin (Figure 2N)	[31]
Heartwood	Ethyl acetate	C-SG	Pterosupin (Figure 2O)	[32]
Heartwood	Aqueous extract	C-SG	Pterocarposide (Figure 2P)	[38]
Heartwood	Aqueous extract	Coulman chromatography over Sephadex LH-20	1-(2′,6′-Dihydroxyphenyl)-β-D-glucopyranoside (Figure 2Q)	[39]
Heartwood	Aqueous extract	C-SG	Pteroisoauroside (Figure 2R) Marsuposide (Figure 3A) Sesquiterpene (Figure 3B)	[30]
Leaves	Methanolic extract	UV-spectrophotometer	Phenolics	[40]
Wood and bark	Ethanolic extract	GC-MS	3-*O*-Methyl-d-glucose (Figure 3C) n-Hexadecanoic acid (Figure 3D) 1,2-Benzenedicarboxylic acid (Figure 3E) Tetradecanoic acid (Figure 3F) 9,12-Octadecadienoic acid (Z,Z) (Figure 3G) D-Friedoolean-14-en-3-one (Figure 3H)	[33]
Apical stem bark	Methanolic extract	Followed standard protocols	Alkaloids Glycosides Flavonoids Terpenoids	[41]
Heartwood	Ethanolic extract	C-SG	Pteroside (Figure 3I) Vijayoside (Figure 3J) *C*-β-D-Glucopyranosyl-2,6-dihydroxyl benzene (Figure 3K)	[42]
Heartwood	Ethanolic extract	C-SG and HPLC	(+)-Dihydrorobinetin (Figure 3L)	[43]
Heartwood	Methanolic extract	LC-MS-MS	Pterosupol Quercetin (Figure 3M) Vanillic acid (Figure 3N) Formononetin (Figure 3O)	[21]
Heartwood	Methanolic extract	HPLC and FTIR	Liquiritigenin	[44]

* Technique—Phytochemical compound identification techniques used, C-SG—Chromatography over Silica Gel, GC-MS—Gas Chromatography-Mass Spectrometry, LC-MS-MS—Liquid Chromatography with Tandem Mass Spectrometry, HPLC—High-Performance Liquid Chromatography, FTIR—Fourier Transform Infrared Spectroscopy.

**Table 2:** Potential activities of some important bioactive compounds or aqueous extracts of *Pterocarpus marsupium* (in chronological order).

S.N.	Extracts/Bioactive Compound	Potential Activities	References
1	(−)-Epicatechin (Figure 2H)	No effect on central nervous system Cardiac stimulant activity Anti-diabetic	[35]
2	Flavonoids	Anti-hyperlipidemic	[31]
3	Phenolics	Anti-hyperglycemic	[32]
4	Pterostilbene (Figure 2A)	Cyclooxygenase-2 (COX-2) inhibition	[52]
5	Pterostilbene and 3,5-hydroxypterostilbene	Induce apoptosis in tumor cells	[53]
6	5,7,2-4 tetrahydroxy isoflavone 6-6 glucoside	Cardiotonic	[45]
7	Pterostilbene	Anti-cancerous Anti-inflammatory Analgesic	[54]
8	Phenolics	Anti-oxidant	[40]
9	Pterostilbene	Anti-cancerous Anti-proliferative	[55]
10	Bark extract	Anti-oxidant Analgesic	[56]
11	Extract of bark and wood	Anti-diabetic Anti-hyperlipidemic	[57]
12	Extract of apical stem bark	Anti-microbicidal	[41]
13	Phenolic-*C*-glycosides	Anti-diabetic	[42]
14	Pterostilbene	Novel telomerase inhibitor	[58]
15	Heartwood extract	Dipeptidyl peptidase-4 (DPP-4) inhibition activity	[59]
16	Heartwood extract	Anti-glycation Sorbitol accumulation Inhibition of aldose reductase	[60]
17	Pterostilbene	Inhibition of platelet aggregation	[61]
18	Heartwood extract	Reduction in body weight Anti-diabetic Anti-hyperlipidemic	[62]
19	(+)-Dihydrorobinetin (Figure 3L)	Radical scavenging activity	[43]
20	Heartwood extract	In vitro lipid lowering activity	[21]
21	Liquiritigenin (Figure 2N)	Hypoglycemic activity	[44]
22	Pterostilbene	Sun (UV rays) protective capacity	[63]

**Table 3:** In vitro propagation protocols for *Pterocarpus marsupium* (in chronological order).

Explants	Source	Media Compositions (Multiplication) *	Culture Response	Media Compositions (Rhizogenesis)	Rooting Response	Plantlets Survival Rate	References
Shoot tip	AS/MT	MS + 0.2 mg·L^−1^ BAP	Ca-Dm	-	-	-	[96]
Aseptic seeds	-	MS basal medium	ISG (95–100%)	-	-	>68%	[26]
Nodal segment	35-d-old-AS	MS + 0.2 mg·L^−1^ IBA	IO	MS + 0.2 mg·L^−1^ IBA	RF		
Cotyledonary node	20-d-old-AS	MS + 4.44 µM BA+ 0.26 µM NAA	SM (85%)	½ MS + 9.84 µM IBA	RF (IVR)	52%	[136]
Cotyledonary node	18-d-old-AS	MS + 5.0 µM BA + 0.25 µM IAA	SM (75%)	2-step-method: PT on ½ MS (liquid) + 200 µM IBA	MRI (40–50%)	-	[25]
				FT on ½ MS (semi-solid) + 0.5 µM IBA	ER (IVR)		
Aseptic seeds	-	½ MS 0.25 mg·L^−1^ GA_3_	ISG (80%)	-	-	-	[98]
Cotyledonary node	18-d-old-AS	2-step-method: SIM: MS + 0.4 µM TDZ	MSI (90%)	2-step-method: PT on ½ MS (liquid) + 200 µM IBA	MRI (65%)	70%	[137]
		FT on SEM: MS + 5.0 µM BA	ES (90%)	FT on ½ MS + 0.5 µM IBA + 3.96 µM PG	ER (IVR)		
Nodal segment	18-d-old-AS	MS + 4.0 µM BA + 0.5 µM IAA + 20 µM AdS	SM (85%)	2-step-method: PT on ½ MS (liquid) + 100 µM IBA + 15.84 µM PG	MRI (70%)	75%	[143]
				FT on ½ MS (semi-solid) + 0.5 µM IBA	ER (IVR)		
Hypocotyl	12-d-old-AS	MS + 5.0 µM 2,4-D + 1.0 µM BA	Ca-Fm (90%)	½ MS + 1.0 µM BA	SEG (56%)	60%	[144]
		MS + 0.5 µM BA + 0.1 µM NAA + 10 µM ABA	SEs (51%)				
Aseptic seed	-	½ MS basal medium	ISG (96%)	-	-	-	[116]
Cotyledonary node	18-d-old-AS	MS + 1.0 mg·L^−1^ BAP + 0.5 mg·L^−1^ NAA	SM (70%)	-	-	-	
Aseptic seed	-	½ MS basal medium	ISG (78.23%)	-	-	-	[19]
Immature zygotic embryo	Green fruits	MS + 3.0 mg·L^−1^ BA + 0.5 mg·L^−1^ IAA	SM (93.8%)	2-step-method: PT on ½ MS (liquid) + 3.0 mg·L^−1^ IBA	MRI (70.8%)	74%	[56]
				FT on ½ MS basal medium	ER (IVR)		
Immature cotyledon	9-d-old-AS	3-step-method: MS + 1.07 µM NAA	Ca-Fm (60.41%)	2-step-method:		95%	[145]
		FT on MS + 8.9 µM BAP + 1.07 µM NAA	MSI (60.41%)	PT on ½ MS (liquid) + 19.6 µM IBA	MRI (75%)		
		FT on MS + 4.4 µM BAP	ES	FT on ½ MS + 2.85 µM IBA	ER (IVR)		
Nodal segment	10-y-old-MT	2-step-method: MS + 13.95 µM Kn + 568 µM AA + 260 µM CA + 605 µM AmS + 217 µM AdS	MSBB (64.44%)	½ MS + 4.92 µM IBA	RF (42%) (IVR)	-	[146]
		FT on MS + 9.3 µM Kn + 0.54 µM NAA + 568 µM AA + 260 µM CA + 605 µM AmS + 217 µM AdS	ES				
Nodal segment	4-w-old-AS	2-step-method: PT on ½ MS (liquid) + 10.0 µM TDZ	MSBB (96%)	2-step-method: PT on ½ MS (liquid) + 150 µM IBA	MRI (80%)	75%	[147]
		FT on MS (semisolid) + 5.0 µM *m*T + 1.0 µM NAA	ES (70%)	FT on ½ MS + 1.5 µM IBA	ER (IVR)		
Cotyledonary node	20-d-old-AS	MS + 7.5 µM *m*T + 1.0 µM NAA	SM (85%)	2-step-method: PT on ½ MS (liquid) + 100 µM IBA	MRI (75%)	80%	[20]
				FT on ½ MS + 1.0 µM IBA	ER (IVR)		
Immature zygotic embryo	Green fruits	MS + 5.37 µM NAA	SEs (67.3%)	½ MS + 5.8 µM GA_3_	SEG (70%)	78%	[148]
		MS + 2.69 µM NAA + 4.4 µM BA + 3% Sucrose					
Shoot tip	7-d-old-AS	MS + 7.0 µM *m*T + 1.0 µM NAA	SM (80%)	2-step-method: PT on ½ MS (liquid) + 250 µM IBA	MRI (67.7%)	96.7%	[149]
				FT on Soilrite	ER (EVR)		
In vitro seedling	Seed	MS + 0.5 µM GA_3_ + 0.5 µM TDZ	SM (85%)	2-step-method: PT on ½ MS (liquid) + 100 µM IBA	MRI (80%)	86.7%	[150]
				FT on ½ MS + 0.5 µM GA_3_	ER (IVR)		
Aseptic seeds	-	½ MS 0.5 µM GA_3_	ISG (91.3%)	-	-	-	[119]

* AA—Ascorbic acid, ABA—Abscisic acid, AdS—Adenine sulphate, AmS—Ammonium sulphate, AS—Axenic seedling, BA—6-benzyladenine, CA—Citric acid, Ca-Dm—Callus-dead mass, Ca-Fm—Callus-fresh mass, d—days, ER—Elongation of roots, ES—Elongation of shoots, EVR—Ex vitro rooting, FT—Followed to transfer, GA_3_—Gibberellic acid, h—hours, IBA—Indole-3-butyric acid, IO—Indirect organogenesis, IVR—In vitro rooting, ISG—In vitro seed germination, Kn—Kinetin, MRI—Multiple root induction, MSBB—Multiple shoot bud break, MSI—Multiple shoot induction, MT—Mature tree, *m*T—*Meta*-topolin, NAA—α-naphthalene acetic acid, PG—Phloroglucinol, PT—Pretreatment, RF—Root formation, SEG—Somatic embryos germination, SEM—Shoot elongation medium, SEs—Somatic embryos, SIM—Shoot induction medium, SM—Shoot multiplication, TB—1,2,3-trihydroxy benzene, TDZ—Thidiazuron, w—week, y—year, 2,4-D—2,4-dichlorophenoxyacetic acid.

## Data Availability

Not applicable.
